# *Quo Vadis* Biomolecular NMR Spectroscopy?

**DOI:** 10.3390/ijms20061278

**Published:** 2019-03-14

**Authors:** Philipp Selenko

**Affiliations:** Weizmann Institute of Science, Department of Biological Regulation, 234 Herzl Street, Rehovot 76100, Israel; philipp.selenko@weizmann.ac.il; Tel.: +972-(0)8-934-2991

**Keywords:** in-cell NMR, time-resolved NMR, post-translational modifications, structure function, intrinsically disordered proteins

## Abstract

In-cell nuclear magnetic resonance (NMR) spectroscopy offers the possibility to study proteins and other biomolecules at atomic resolution directly in cells. As such, it provides compelling means to complement existing tools in cellular structural biology. Given the dominance of electron microscopy (EM)-based methods in current structure determination routines, I share my personal view about the role of biomolecular NMR spectroscopy in the aftermath of the revolution in resolution. Specifically, I focus on spin-off applications that in-cell NMR has helped to develop and how they may provide broader and more generally applicable routes for future NMR investigations. I discuss the use of ‘static’ and time-resolved solution NMR spectroscopy to detect post-translational protein modifications (PTMs) and to investigate structural consequences that occur in their response. I argue that available examples vindicate the need for collective and systematic efforts to determine post-translationally modified protein structures in the future. Furthermore, I explain my reasoning behind a Quinary Structure Assessment (QSA) initiative to interrogate cellular effects on protein dynamics and transient interactions present in physiological environments.

## 1. Preface I

In-cell nuclear magnetic resonance (NMR) spectroscopy has become increasingly popular amongst the biomolecular NMR community, especially in funding statements regarding the versatility of general NMR methods or to embellish visionary outlooks in grant applications of high-risk caliber. In practice, genuine in-cell NMR measurements remain sparse and few laboratories are committed to performing these experiments. As a consequence, primary in-cell NMR work is all too often outnumbered by recurring reviews [1,2,3]. To not perpetuate this trend, I refrain from discussing in-cell NMR work itself but rather focus on spin-off applications whose developments were stimulated by cellular NMR approaches. As we shall see, these alternatives offer enticing possibilities for the biologically inclined NMR spectroscopist, especially in light of the resolution revolution in cryo-electron microscopy (EM) and its impact on all areas of structural biology [4].

## 2. Preface II

The arrival of cryo-EM in the realm of genuine atomic-resolution methods has changed the structural biology landscape in a most profound manner. While X-ray crystallographers adapted swiftly to this new reality, largely by abandoning their methodological preferences altogether, the biomolecular NMR community has been slow in accepting what is evidently more than a fleeting trend. Without a doubt, the future of structural biology will be shaped by EM-based methods with recent breakthroughs offering a mere glimpse into the great potential of the technique. This holds true for advancements in EM technologies, as well as for turning previously intractable biological questions into feasible research projects. By agreeing that EM will dominate the field of structural biology in the next decades, we may ask what questions the community will address with it? Obvious answers include large biomolecular complexes and machines, reconstituted and native membrane proteins, macromolecular protein assemblies including oligomers, fibrils and ordered aggregates, especially those purified from natural sources, as well as low abundance specimens and samples [5]. While such targets may initially be chosen based on their structural rigidity and size, these limitations will likely shift to smaller scales and more flexible assemblies in the near future. Beyond these self-evident trends, another popular theme in general biology is also making its mark in structural biology—the goal to investigate biomolecules where they naturally occur, that is, inside cells. In most cases, this means to derive structural and functional insights directly in intact cellular specimens. Accordingly, the term cellular structural biology was coined to collectively describe such high-resolution in situ efforts [1,6,7,8]. X-ray crystallography is mostly excluded from cellular applications due to the need for crystalline samples, which are (almost) impossible to obtain inside cells [9]. By contrast, the history of electron microscopy is firmly rooted in cellular investigations and precedes high-resolution structural studies by decades. Concomitantly, technical developments in cell- versus molecule-based EM methods ran parallel for many years and converged only recently. This was largely due to technical advancements including direct electron detectors, fast imaging modalities and computational ‘drift’ corrections, improved energy filters, and phase-plate technologies for greater contrast [10,11]. These developments offered mutual benefits to both branches of EM methods by alleviating common bottlenecks. Today’s breakthroughs in cellular cryo-electron tomography (CET) [12,13,14], cryo-scanning transmission electron tomography (CSTET) [15,16], and correlative light and electron microscopy (CLEM) [17,18,19] may seem synonymous with advances in single particle cryo-EM, although, strictly speaking, they developed independently. CET, CSTET, and CLEM represent the core tools of current EM methods in cellular structural biology, with most insights generated by CET of vitrified prokaryotic [20] or focused-ion beam (FIB) milled eukaryotic cells [21], commonly by placing previously determined, high-resolution structures into subtomogram-averaged densities [22]. This will likely change in the future and three-dimensional architectures of proteins and their complexes will be solved directly in cells. These outlooks serve as the preamble for all structural in situ studies today, including those by in-cell NMR spectroscopy. In comparison, past, present, and future in-cell NMR efforts are at a sizable disadvantage.

## 3. Pitfalls, Challenges, and Opportunities

Let me first survey the historical context of modern biomolecular NMR applications and their standing in the aftermath of the EM revolution in resolution. Throughout the 1990s, and following the discovery of independently folded protein domains as key functional units in many biological processes, NMR spectroscopy and X-ray crystallography often pursued these targets in a friendly competitive manner [23,24]. Based on the growing awareness that protein domain functions can only be appreciated in their extended modular contexts, ever more sophisticated NMR approaches, often combined with complementary techniques such as small-angle X-ray scattering (SAXS) aimed at determining structures of large multi-domain constructs [25]. Faced with the dilemma of exponential signal multiplication with target size and correspondingly adverse relaxation properties, the biomolecular NMR community devised several ingenious ways to deal with such samples. These include (transverse) relaxation-optimized spectroscopy (TROSY) [26,27], amino acid-specific, site-selective, and segmental isotope labeling [28], higher-dimensionality NMR experiments [29], and non-uniform sampling schemes [30]. Despite these developments, structure determination efforts of large, folded proteins by solution-state NMR spectroscopy remained challenging and cumbersome. Luckily, the emergence of structural disorder as a fundamental property of higher eukaryotic proteomes presented itself as the timely savior from this failing arms race [31,32]. Members of the biomolecular solution NMR community, including us, converted to the safe haven of intrinsically disordered proteins (IDPs) in large numbers and with great fervor [33,34,35,36]. In the absence of natural predators, solution NMR flourished in its newly discovered habitat and, emboldened by previous advancements that proved equally if not more useful in the world of dynamic disorder, it experienced a formidable revival [37]. Nonetheless, some conceptual problems persisted, which, I believe, were often rooted in the classically trained mindsets of its ‘structured’ protagonists and a certain negligence of the biological implications of disorder with regard to function rather than to structure (or absence thereof). By yet another stroke of good luck, liquid–liquid phase separation and membraneless organelles emerged as the timely saviors of IDP–NMR scientists in the midst of their structure–function identity crises [38,39]. Suddenly, the absence of structure became ‘function’ and a thorough discussion about the general role of disorder in biological systems could safely be postponed.

In parallel to these topical developments, solid-state (ss) NMR spectroscopy matured into a veritable contestant for structural analyses of high-molecular-weight assemblies of ordered proteins, including viral particles [40], amyloid fibrils [41], and membrane proteins [42]. Emboldened by similarly brazen developments in ssNMR methods, including proton-based excitation and detection routines [43], dynamic nuclear polarization (DNP) [44], and probes for magic angle spinning (MAS) at ultra-fast frequencies (>100 kHz) [40], biological ssNMR applications reached unprecedented levels of sophistication. However, many high-quality ssNMR samples were equally good cryo-EM specimens, which led to both methods often pursuing the same targets. This is illustrated by complementary ssNMR and cryo-EM studies on amyloid proteins such as Het-S [45,46,47,48,49], amyloid-β [50,51,52,53], α-synuclein [54,55,56,57,58,59,60,61,62] and tau [63,64,65,66,67,68], viral particles [69,70,71], membrane proteins including bacterial secretion systems [72,73,74,75,76], and transporters [77,78,79]. Once again, the competitive outcomes of these efforts proved unfavorable for NMR spectroscopy, especially in terms of cost effectiveness, required sample types and quantities, experimental times, and computational efforts to turn measured distance restraints into high-resolution, structural models. In addition, ssNMR often provided restricted information about parts of these multicomponent machineries, whereas cryo-EM revealed comprehensive insights into entire assemblies. Moreover, CET and cryo-EM methods were beginning to resolve these structures in their native cellular settings, as recently shown for the type III secretion system [80,81] or with specimens purified directly from post-mortem brains as in the case of human tau filaments [66,67,68], respectively. These breathtaking developments consolidated a similarly critical assessment of solid-state NMR spectroscopy by many structural biology departments turning to cryo-EM as their preferred option for future investments instead.

In-cell NMR spectroscopy entered this stage about two decades ago [82]. Despite the largely benevolent patronage of the biomolecular NMR community, it developed slowly even though it offered a principally attractive means to populate a structural biology niche that no other method could claim at that time: The ability to obtain atomic-resolution insights into protein, RNA, and DNA structures directly in live cells. While in-cell NMR was nourished with good intentions, most biomolecular NMR laboratories chose to observe it from a distance rather than getting involved themselves. After all, those were the times of NMR resources and projects abound and dabbling in unknown territories such as cells required skills that were too remote for many to bother with. As a consequence, the early days of in-cell NMR were spent in comfortable solitude. Over the years, and having transcended from bacteria to higher eukaryotic and mammalian cells, interest within the community grew. Encouraged by more frequent high-impact publications and dedicated in-cell NMR sessions at regular NMR meetings, additional groups began to explore the method. With times changing, cryo-EM looming, and NMR resources dwindling, the prospects of in-cell NMR, including the comfort of a protected niche, are becoming ever more appealing. Despite this positive trend, true in-cell NMR applications remain challenging and initial experiments often fail to produce the desired outcomes (also for reasons that recent in-cell NMR work has helped to elucidate, see below). To minimize such drawbacks, I advise novice users to follow three simple rules when embarking on in-cell NMR adventures. First, start with crude lysates of your ‘empty’ host cells and add isotope-labeled proteins/biomolecules directly to these slurries for pilot NMR experiments. Possible interactions with cellular components will be recapitulated in these mixtures and degrees of (site-selective) line broadening will be indicative of scenarios to be encountered in cells (see also the quinary structure section at the end of this text). Second, set up stringent control experiments to ensure that enrichment or delivery of isotope-labeled biomolecules indeed produces cellular samples that harbor the species of interest. Third, maximize efforts to quantify the amount of target biomolecules in these samples in order to (a) repeat lysate experiments at relevant (low μM) concentrations and (b) optimize enrichment or delivery procedures for satisfactory signal-to-noise ratios within realistic data acquisition times. Guidelines for how to measure intracellular concentrations of delivered/enriched biomolecules in cells are provided in several published protocols [83,84,85,86]. Together, these steps will allow newcomers to pre-assess the overall feasibility of in-cell NMR projects.

As I said before, the goal of this review is not to discuss in-cell NMR applications but to focus on concepts and approaches that the method helped to popularize. Similar to in-cell NMR, these are principally rooted in five fundamental aspects of NMR spectroscopy: One, NMR is an atomic-resolution method. Two, NMR is quantitative in that signals reflect the number of NMR-active nuclei in the sample. Three, NMR resonance frequencies (chemical shifts) are uniquely sensitive to the chemical environment of active nuclei, which provides structural and functional information. Four, NMR signals contain additional information about the dynamic properties of observable spin systems. Five, NMR spectroscopy is a non-destructive method and works at physiological temperature and pH. As we shall see next, this minimal set of key NMR properties provides the basis for genuine applications off the paths of conventional structural biology routines.

## 4. Detecting Post-Translational Protein Modifications by NMR

In cells, most proteins undergo different post-translational modifications that alter the chemical identities of individual residues [87]. The establishment of PTMs is strictly regulated and mediated by dedicated sets of enzymes, whose own activities are often controlled by PTMs. Together they form cascading feedback networks of interconnected signaling pathways. Importantly, most PTMs constitute reversible ‘switches’ that reprogram the functions of proteins in response to external and internal cellular cues. Therefore, they serve as key regulators in virtually all processes of life. PTMs typically occur at multiple sites and in disordered protein regions [88,89]. Their addition and removal are highly dynamic and combinatorial modification patterns are often established in multistep reaction mechanisms with clearly defined hierarchies [90]. Given the immense importance of PTMs in modulating protein functions, biologists seek to understand where PTMs occur, which types of modifications are present, and in what combinations, how they are established (and removed), and how they impact the biological activities of target proteins. The breadth of these questions explains the great need for analytical tools to annotate and investigate protein PTM states.

The unique sensitivity of the NMR chemical shift on its immediate chemical environment provides an exquisite readout modality for PTM states of individual protein residues. In other words, changes in the chemical composition of protein side-chain moieties, as imposed by different PTMs, result in chemical shift changes that are characteristic of the respective type of modification (see [91] for a comprehensive overview). In turn, differences in protein–NMR spectra reveal the sites of PTMs as well as their nature, which constitutes a highly attractive, analytical feature. In 2008, we reported how in-cell NMR can be used to detect and site-specifically assign cellular protein phosphorylation events, the most common type of eukaryotic PTM [92]. While our publication was not the first to describe the detection of protein phosphorylation by NMR spectroscopy, and was clearly inspired by earlier work by Lippens, Gronenborn, Forman-Kay, and others [93,94,95], it probably boosted renewed interest in using NMR to decipher more complex modification reactions and their underlying mechanisms. One aspect of our paper that may have additionally stimulated this trend was the use of cell lysates containing native cellular enzymes and modification reactions that we carried out directly in these lysates, thus offering a cost-effective and convenient way to phosphorylate isotope-labeled proteins for NMR measurements in vitro and in situ [96]. In the following years, the rationale for detecting protein phosphorylation by NMR spectroscopy was successfully employed by many groups (see below). Importantly, most of these studies were aimed at deriving functional insights rather than at delineating structural information about modified substrate states. By exploiting the non-destructive and quantitative nature of such NMR measurements, we illustrated another analytical advantage of this approach: The ability to directly follow PTM reactions in a time-resolved fashion in order to deduce site-specific modification rates [97,98]. Indeed, protein phosphorylation studies by time-resolved NMR spectroscopy became very popular and proved essential for delineating mechanistic insights into diverse sets of signaling reactions [99,100,101,102,103,104,105,106,107,108,109,110,111,112,113,114,115,116,117,118,119,120,121,122,123,124,125,126,127,128,129,130,131,132,133,134,135].

As stated earlier, NMR detects PTMs irrespective of their nature. Moreover, different PTM chemistries impose characteristic spectral signatures, which serve to identify the corresponding modification type(s) [91]. Therefore, functional studies of protein phosphorylation represent just one aspect of NMR’s power as an analytical tool. Indeed, several other protein PTMs have been analyzed by NMR spectroscopy, including acylation, alkylation, and glycosylation [136,137,138]. More recently, we used time-resolved NMR spectroscopy to study methionine oxidation as an example of a non-enzyme mediated protein PTM, which occurs frequently in response to oxidative cell stress and organismal aging [139]. Specifically, we monitored the site-selective repair of the oxidation-damaged α-synuclein by endogenous cellular enzymes. Differences in NMR chemical shifts of native and oxidation-damaged proteins allowed us to follow individual repair reactions in a time-resolved manner with single-residue resolution. Similarly, we performed time-resolved in situ NMR measurements to monitor irreversible protein cleavage in isolated protein–protease mixtures, cell lysates, and intact cells [140]. Together, these examples underscore the great potential of NMR-based PTM studies in reconstituted in vitro systems ranging from defined enzyme-substrate mixtures to native, cell-free lysates. They also paved the way for applications that I will discuss next: NMR investigations of changes in protein structures upon post-translation modifications.

## 5. Making and Breaking of Protein Structures by PTMs

In the previous paragraph, I outlined how NMR spectroscopy can be employed to functionally annotate different types of PTMs in a residue- and time-resolved, quantitative manner. While such applications exploit the analytical power of NMR spectroscopy, they offer an attractive additional feature: The ability to correlate the establishment of different PTMs with structural alterations that occur in their response. Phosphorylation in particular has long been known for its capacity to alter protein structures [141], especially by strengthening or weakening secondary structure elements such as α-helices in a position-dependent manner [142]. Whereas phosphorylation of N-terminal helix residues stabilizes helicity via capping interactions, modifications at C-terminal helix residues add to the negative dipole moment at helix ends and act destabilizing [143]. Many examples of such stabilizing and destabilizing phosphorylation effects in classically folded proteins are known [144]. The emergence of IDPs has helped to extend this concept to regions of residual secondary structure, where phosphorylation appears to exert even greater effects [93,108,145]. Given that pre-structured motifs usually function as molecular recognition elements in IDP–ligand interactions [146], signaling and phosphorylation-dependent changes in these structural propensities influence binding energies and affinities in pronounced ways, especially in binding-induced disorder-to-order and order-to-disorder transitions [147]. Systematic structural investigations into these types of IDP–PTM interactions are scarce [148], although I believe that they provide important new insights into the roles of PTMs in signaling-mediated structure–function relationships. At this point, I wish to reiterate that solution NMR is uniquely capable of providing this information, especially with regard to dynamic and partially disordered protein regions, where most eukaryotic PTMs occur. No other atomic-resolution method can unravel these scenarios at comparable levels of resolution and with similar ease. Indeed, several publications pay tribute to the great power of NMR in such structure–function analyses [94,100,105,149,150,151,152,153,154,155,156,157,158,159]. In the following paragraphs, I discuss three examples that illustrate the scope of phosphorylation-induced structural rearrangements and NMR’s excellent ability to decipher them, and their resulting architectures.

My first example is the human splicing factor 1 (SF1). Together with the large and small subunits of the U2 small nuclear ribonucleoprotein auxiliary factor (U2AF), SF1 defines the 3′ splice site recognition complex on pre-messenger RNA. While SF1-U2AF binding is primarily mediated by a canonical tryptophan–RNA recognition motif (RRM) interaction [160], the ternary RNA–protein complex is further stabilized by SF1 phosphorylation at two adjacent residues [161]. In 2013, two groups independently reported the molecular basis for this behavior by determining the X-ray and NMR structures of phosphorylated and unmodified SF1-U2AF complexes [162,163]. The main reason for the importance of these publications is that they provide complementary information about the structural effects of SF1 phosphorylation that are inaccessible to either experimental method alone. Hence, in this case, the combination of X-ray crystallography and NMR spectroscopy revealed the full scale of the phospho-regulation of the SF1-U2AF interaction. Residues in the N-terminus of SF1 arrange in a helix-hairpin conformation with a flexible ~30-residue linker connecting the two α-helices. In unmodified SF1, this intrinsically disordered region (IDR) exhibits high internal dynamics and samples a range of conformations [163]. In turn, the IDR of unmodified SF1 is poorly defined by X-ray crystallography with missing electron density for most of its residues [162]. By contrast, the very same residues were perfectly tractable by NMR and their dynamic properties annotated with high precision [163]. Upon phosphorylation of the two SF1 serines, the IDR becomes fixed in a rigid conformation, primarily via coordination of the two phosphate moieties by conserved arginines at the N-terminus of the second SF1 helix. Accordingly, the X-ray structure provided detailed insights into the coordination of phosphate oxygens by side-chain guanidium groups in a tight arginine ‘claw’ [162]. NMR spectroscopy, on the other hand, measured greatly reduced dynamics of the phosphorylated linker and the formation of a stable, albeit disordered structure [163]. Thus, the combination of both methods revealed how dual phosphorylation of two SF1 linker sites locked the IDR in a conformation that cooperatively enhanced U2AF binding via reducing the entropic penalty of the encounter complex. The SF1-U2AF example provides several important lessons: First, it outlines how the regulated establishment of intramolecular IDR contacts can drastically alter the binding behavior of two proteins. Second, it illustrates how cell signaling and protein phosphorylation regulate this behavior in a fully reversible manner. Third, it underscores the importance of novel types of intramolecular coordination chemistries between PTMs and protein residues in forming previously unknown structures such as the arginine–phosphate claw observed here. Given the abundance of protein phosphorylation throughout eukaryotic biology, such reversible PTM structures likely constitute common themes in many signaling processes. Intramolecular arginine–phosphate contacts, for example, are present in a large number of substrates and clearly qualify as a general structural principle [159,164,165]. Despite this, only a handful of PTM motifs are known to adopt defined conformations in their modified states [166], with kinase domains and their phosphorylated activation loops serving as the most prominent examples [167]. Accordingly, our understanding about sequence features giving rise to such structures is limited and we are unable to predict when and where they occur, or what types of conformations they adopt. Therefore, I believe that the conformational space of possible three-dimensional protein ‘folds’ is much larger than we think and that we will only grasp its full dimensions when we begin to analyze the structures of post-translationally modified proteins in a systematic manner. Given that disorder and high degrees of protein flexibility will likely prevail in these uncharted territories, NMR spectroscopy is ideally suited to uncover novel PTM structures.

Along those lines, I discuss here another extraordinary example of PTM-induced protein folding: The eukaryotic translational initiation factor 4E (eIF4E) binding protein, 4E-BP. In its non-phosphorylated form, isolated 4E-BP is fully disordered [168,169]. In the presence of eIF4E, 4E-BP adopts a helical conformation and binds eIF4E via a conserved, hydrophobic interaction motif [170]. Surprisingly, phosphorylated 4E-BP fails to interact with eIF4E [171]. In 2015, a concerted NMR effort revealed stunning insights into the mechanistic basis for this behavior. Kay and Forman-Kay, et al. showed that signaling-mediated modification of 4E-BP at two threonine residues upstream the hydrophobic interaction motif induced complete folding of the eIF4E cognate site into a binding-incompetent β-sheet structure [172]. Thus, phosphorylation of 4E-BP switches the protein between a disordered and a β-strand conformation, in which the eIF4E binding site is inaccessible. By doing so, 4E-BP defines a functional signaling mechanism that is entirely different in its mode of action. Rather than to establish a recruitment platform for phospho-binding proteins, phosphorylation masks an existing interaction motif by virtue of incorporating it into a folded structure. Therefore, the 4E-BP example of phosphorylation-induced protein folding adds a novel structural dimension to eukaryotic signaling processes. Probably the most radical aspect of this and the previously discussed SF1 case is the direct involvement of phosphates in forming the core contacts of the newly formed structures [162,172]. Both examples reveal radical new principles of how phosphate coordination gives rise to globular protein folds without classical hydrophobic cores [173]. Built from disorder, these structures also suggest new paradigms for biological regulation. Phosphorylation-induced protein folding may shield existing binding sites, or create new ones. It may expose critical residues for ubiquitination and, thereby, trigger cellular degradation, or act in the opposite direction and prevent protein turnover, thus extending the lifetimes of molecular players. Most importantly, SF1 and 4E-BP exemplify new modes of biological regulation and remind us that many of them remain to be discovered. In addition, both provide compelling testimonies to the power(s) of NMR in deciphering such complex structure–function relationships.

Finally, I outline an example in which phosphorylation triggers the opposite effect in that it unfolds a folded protein domain [174,175]. Specifically, I discuss how phosphorylation of the human cell-cycle regulator and cyclin-dependent kinase inhibitor p19^INK4d^ drives the transition from G1 to S-phase in an irreversible manner [176]. In contrast to phosphorylation-induced folding of 4E-BP, dual phosphorylation of p19^INK4d^ acts to dissolve its central ankyrin-repeat domain in a stepwise manner. While modification of the first residue destabilizes N-terminal helices to provide access for a second enzyme to phosphorylate a previously inaccessible site, establishment of both modifications unfolds the entire N-terminus of p19^INK4d^. This exposes conserved lysine residues for ubiquitination, which, in turn, triggers p19^INK4d^ degradation by the proteasome and clearance from cells. At the same time, the two-step cascade also abolishes the structured CDK6 binding interface on p19^INK4d^, which disrupts the inhibitory CDK6-p19^INK4d^ complex, releases CDK6, and activates it to signal G1/S transition. Thus, p19^INK4d^ phosphorylation, unfolding, and degradation collectively act to ensure the directionality of cell-cycle progression, probably one of the most important processes in eukaryotic biology. The beauty of this mechanism, and the corresponding study, lies in the exquisite combination of NMR spectroscopy and cell biology methods, which, I believe, serve as a paradigm for future investigations in these directions. NMR experiments were performed with isolated, recombinant enzymes as well as in lysates of cells arrested at different stages of the cell cycle and containing different sets of active, endogenous kinases. Structure–function analyses combined with biochemical pull-down and protein detection assays revealed the phosphorylation-triggered dissociation of the CDK6-p19^INK4d^ complex, subsequent ubiquitination, and cellular clearance of p19^INK4d^. All in all, a benchmark study of how biomolecular NMR spectroscopy may be used in combination with complementary biochemistry techniques to reveal novel modes of biological regulation. The overall paucity of PTM-induced folding and unfolding examples may have two reasons: Either they are rare, or we have not used adequate tools to uncover them on a broader scale. I am convinced of the latter and believe that NMR spectroscopy can serve as a key discovery technique in these investigations.

## 6. Physiological Protein Dynamics and Quinary Structure

Finally, I want to touch upon a subject that I feel is underrepresented in NMR studies aimed at resolving physiological protein behaviors. On the one hand, NMR is uniquely capable of providing quantitative information about protein dynamics over time scales spanning several orders of magnitude, from pico-seconds (10^−12^ s) to hours (~10^4^ s) [177,178,179]. On the other hand, most NMR relaxation measurements are performed on isolated, dilute samples that bear little resemblance to the cellular environments where proteins function [180]. As a result, we gathered a wealth of information about protein dynamics in artificial in vitro settings without understanding how they are manifested in vivo. I believe that this has led to several misconceptions about relevant time scales of protein motions in cells [37]. I agree that comprehensive NMR relaxation studies under true in-cell conditions are difficult to perform. I also acknowledge that in vitro settings that approximate cellular environments often fail to recapitulate physiological protein behaviors [181]. One reason for this shortcoming lies in the overwhelming complexity of the intracellular milieu, both in terms of composition and organization. Accordingly, in cells, proteins experience highly diverse sets of encounters and continuously engage in transient, low-affinity associations that may outnumber specific binding events in abundance and frequency. These interactions are often protein and cell type-specific and appear to have been evolutionarily optimized across entire proteomes [182,183]. Despite the randomness of these short-lived encounters, their configurations, orientations, and binding surfaces tend to deviate from stochastic behaviors. Specifically, properties such as charge distributions and local hydrophobicity steer association characteristics that are not uniform across protein surfaces [184]. As a result, some areas display higher propensities for unspecific binding events, or, more accurately, they mediate interactions of extended residence lifetimes. With respect to protein dynamics, this results in non-uniform attenuations that cannot be recapitulated with single crowding or viscosity agents. The nature of these effects in cells, both specific and unspecific, and their combined influence on protein structure and dynamics is summarized in the term “quinary protein structure”—in extension to the classical definition of primary, secondary, tertiary, and quaternary protein structures. While quinary protein structure may be considered a modern concept, it was first coined in the 1980s without much traction for the remainder of the century because tools to assess quinary structure in cells did not exist [185,186,187]. Modern in situ methods including in-cell Förster resonance energy transfer (FRET) and NMR spectroscopy helped to change this notion, and contributed to a formidable revival of the term, with several studies emphasizing its rediscovered biological importance [102,183,188,189,190,191,192,193,194,195]. One general conclusion from these investigations is that intracellular environments and respective quinary structure interactions can have opposite effects and may act stabilizing or destabilizing in a protein-specific manner [196]. Another observation that we and others made is that partially structured motifs and regions of residual structure, synonymous with protein interaction sites, are usually more prone to exhibit quinary structure effects than biologically inert parts of proteins, as expected for functional but uncomplemented electrostatic and hydrophobic surfaces [197]. In turn, quinary interactions at these sites attenuate NMR relaxation properties and respective signal qualities to greater extents than cellular viscosity and crowding alone. Such effects serve as useful indicators for regions of biological interest and their identification may aid the discovery of new interaction hotspots even when the types and identities of interacting ligands are not known. Given the great predictive value of this information, I propose to launch a concerted *Quinary Structure Assessment (QSA)* initiative to annotate the impact of intracellular environments on protein structures and dynamics. To do so, I invite interested NMR groups to record 2D ^1^H-^15^N NMR spectra of their favorite proteins in a standard buffer and in easily accessible cell lysates prepared from bacteria (*E. coli*) [198] and a mammalian cell line such as HeLa (*H. sapiens*) [98]. By acquiring reference and lysate NMR spectra with identical spectrometer settings, they will be able to extract information about site-selective line broadening. Even when resonance assignments are not available, such comparisons will provide qualitative and quantitative information about altered relaxation properties due to quinary structure interactions. Sharing these results with dedicated in-cell NMR laboratories such as ours will allow us to formulate multi-component mixtures containing metabolites, RNA, DNA, proteins, and lipids, in order to reproduce the observed cellular effects under stable in vitro conditions. Stock solutions of these mixtures may then be distributed amongst the biomolecular NMR community to collectively probe quinary structure interactions in other protein samples, which will reveal fundamental insights into core aspects of cellular biology and biophysics. As an added value, they will provide general feasibility assessments for in-cell NMR approaches with individual target proteins (see recommendations for pilot in-cell experiments above).

## 7. Conclusions

In summary, I have presented a collective outlook towards the implementation of biomolecular NMR methods to investigate basic biological processes under experimental conditions that (a) approximate native cellular settings, (b) exploit endogenous machineries such as enzymes, and (c) follow their activities in a time-resolved and quantitative fashion. Specifically, I outlined the use of solution NMR spectroscopy to study post-translational protein modifications (PTMs) and their effects on protein structure and dynamics, and to perform time-resolved NMR analyses of enzyme-substrate reactions to determine modification rates, processing mechanisms, as well as PTM hierarchies and cross-talks. Furthermore, I introduced quinary structure interactions and how they affect in-cell protein dynamics. Importantly, most of the methodological aspects discussed in this review concern NMR experiments performed outside of cells, with standard protein samples, spectrometer setups, and settings. As I hope to have conveyed to the reader, I firmly believe that these NMR applications offer exciting new research directions beyond classical structure determination routines. In this day and age, we, the biomolecular NMR community, are well advised to explore these non-conventional paths with vigor, rigor, and timely urgency.

## Abbreviations

NMR	Nuclear Magnetic Resonance
EM	Electron Microscopy
CET	Cryo-Electron Tomography
CSTET	Cryo-Scanning Transmission Electron Tomography
CLEM	Correlative Light and Electron Microscopy
TROSY	Transverse Relaxation Optimized Spectroscopy
MAS	Magic Angle Spinning
DNP	Dynamic Nuclear Polarization
